# Chinese patent herbal medicine Huaiqihuang for Henoch-Schonlein purpura nephritis in children: a systematic review of randomized controlled trials

**DOI:** 10.1186/s12906-021-03415-x

**Published:** 2021-11-08

**Authors:** Xue Xue, Xue-han Liu, Chun-li Lu, Xin-yan Jin, Qiang Liu, Xiao-qin Wang, Jian-ping Liu

**Affiliations:** 1 Hubei University of Traditional Chinese Medicine, The first clinical college and affiliated hospital, Wuhan, 430061 Hubei China; 2 Beijing University of Chinese Medicine, Centre for Evidence-Based Chinese Medicine, Beijing, 100029 China

**Keywords:** Chinese patent herbal medicine, Huaiqihuang, Henoch-Schonlein purpura nephritis, Children, Randomized controlled trials, Systematic review

## Abstract

**Background:**

Henoch-Schönlein purpura nephritis (HSPN) is listed as the most common secondary glomerular diseases among children. Approximately 15 to 20% of children eventually could develop into chronic renal failure. Chinese patent herbal medicine Huaiqihuang (HQH) has been widely used in children with HSPN. This study aimed to evaluate the effectiveness and safety of HQH for HSPN in children, so as to provide evidence for clinical use.

**Methods:**

Randomized controlled trials (RCTs) on HQH for HSPN in children were searched in eight Chinese and English databases from their inception to December 2020. We included children with HSPN received HQH combined with conventional medicine. Cochrane “Risk of bias” tool was used to assess methodological quality, and “Grading of Recommendations Assessment, Development, and Evaluation (GRADE) approach” to summarize the certainty of evidence for main findings. Effect estimates were presented as risk ratio (RR), mean difference (MD) or standardized mean difference (SMD) with 95% confidence interval (CI) in meta-analyses using RevMan 5.3. Data not suitable for statistical pooling were synthesized qualitatively.

**Results:**

In total seven RCTs were identified. Compared with conventional medicine alone, HQH plus conventional medicine showed the better effect in improving clinical cure rate (RR 1.58; 95%CI 1.17 to 2.14; *n* = 6) and total effective rate (RR 1.34; 1.16 to 1.54; *n* = 6); reducing urine sediment erythrocyte count (MD -9.23; − 10.76 to − 7.69; *n* = 3) and urine β2 micro-globulin level (MD -0.09; − 0.12 to − 0.06; *n* = 2). No serious adverse event was recorded in all included trials.

**Conclusions:**

Limited evidence showed HQH combined with conventional medicine had a beneficial effect for children with HSPN, and the side effects were mild. HQH may be a promising complementary therapy. However, long term follow-up, high quality and multicenter RCTs are required to confirm the findings.

## Background

Henoch-Schönlein purpura (HSP) is an immunoglobulin A mediated disease characterized by a generalized vasculitis mainly involving the skin, joints, gastrointestinal tract, and kidneys [1]. Among them, HSP nephritis (HSPN) is potentially the most severe complication [2], which usually manifests as gross hematuria, microscopic hematuria, microalbuminuria to massive proteinuria, and even renal failure [3]. A national cross-sectional survey in China reported that HSPN was listed as the most common secondary glomerular diseases among children [4]. Although most children with HSPN have a good chance of achieving a recovery, some may have a prolonged course of disease, approximately 15 to 20% of children will eventually develop chronic renal failure [5]. Thus, it is of great importance to pay enough attention to it.

There is no unified treatment plan for HSPN because of the diverse clinical manifestations and complex pathological classification of HSPN [5]. At present, the treatment mainly includes symptomatic treatment, angiotensin-converting enzyme inhibitors (ACEIs) or angiotensin receptor blockers (ARBs), glucocorticoid (GC) and immunosuppressant (IS) [6, 7]. However, the effectiveness is limited, and it is easy to relapse after discontinuation of medicine. Long-term use of GC and IS may cause serious side effects and toxicity. Consequently, it is necessary to seek alternative and supplementary therapies to treat HSPN in children in order to improve disease remission rate, reduce relapse and relieve side effects.

Huaiqihuang (HQH) granule is a Chinese patent herbal medicine which contains Huaier, Gouqizi and Huangjing. All ingredients of HQH were shown in Table 1. All of these have been extensively used in China for thousands of years [8]. In recent years, HQH granules have been widely applied in the clinical treatment of various chronic kidney disease, including primary nephrotic syndrome, IgA nephropathy, HSPN and other primary and secondary kidney diseases [9–13]. Among them, a number of clinical trials have shown that HQH combined with conventional medicine has a significant effect on reducing proteinuria, mitigating hematuria, preventing relapse and relieving clinical symptoms in children with HSPN [13–19]. Combined with the results of animal studies and in vitro experiments, the clinical effectiveness of HQH may be related to a variety of mechanisms, including regulating immune response, reducing inflammatory damage, protecting podocytes, lightening the proliferation of glomerular mesangial cells and improving renal fibrosis [20–25]. Currently, only some small sample clinical trials have reported the effect of HQH on children with HSPN, and there is still a lack of systematic reviews on its overall effect and safety. As a result, this review aimed to review systematically and evaluate the effectiveness and safety of HQH on treating pediatric HSPN based on randomized controlled trials so as to provide statistically reliable evidence.

**Table 1:** Ingredients of Huaiqihuang granule

Latin binomial name	Pinyin	Chinese name
*Trametes robiniophila Murr*	Huaier	槐耳
*Lycium barbarum*	Gouqizi	枸杞子
*Polygonati sibiricum*	Huangjing	黄精

## Methods

### Protocol and registration

Methods of this review were specified in advance and documented in International Platform of Registered Systematic Review and Meta-analysis Protocols (INPLASY.COM) on 31th of December 2020. (Registration number: 2020120148). The content of review was reported according to the Preferred Reporting Items for Systematic Reviews and Meta-Analyses (PRISMA-2009) [26].

### Eligibility criteria

#### Types of studies

Randomized controlled trials (RCTs) regardless of the blinding method were identified.

#### Types of participants

All children with HSPN under 14 years old were included. No restriction on sex or race of participants. HSPN diagnosis was made according to Clinical Guidelines-a division of nephrology edited by Pediatrics Branch of Chinese Medical Association in 2009 [27]. That was, within 6 months of the course of HSP, hematuria and/or proteinuria appeared, with or without hypertension, edema, and renal damage. “Hematuria” included gross hematuria or microscopic hematuria. Besides, “proteinuria” need meet any of the following: (1) urine routine protein was positive within 1 week for 3 times; (2) 24 h urine protein quantitative> 150 mg; (3) urine microalbumin was higher than the upper limit of normal value within 1 week for 3 times. The pathological diagnosis of HSPN referred to the classification of the International Study of Kidney Disease in Children (ISKDC), and was divided into I-VI grades [27].

#### Types of intervention

In addition to basic treatment (including: diet management, avoidance of allergens, anti-infection, anti-platelet aggregation, anti-allergy, ACEI / ARB, and other sympto-matic treatments.), experimental group took “HQH” or “HQH combined with GC” or “HQH combined with GC and IS” treatments. While control group was administered “GC” or “GC combined with IS” on the basis of basic treatment (BT).

#### Types of outcome measures

Primary outcome measures: (1) clinical cure rate; (2) total effective rate. Secondary outcome measures: (1) 24 h urinary protein excretion; (2) urine sediment erythrocyte count; (3) urine β2 micro-globulin(β2-MG); (4) immune cells and inflammatory factors; (5) adverse events.

“Clinical cure” referred to the disappearance of symptoms and signs(purpura of the skin, joint swelling and pain, melena, hematuria, edema, hypertension.); urinary protein and urinary sediment erythrocyte continued to turn negative; or urinary protein quantitative< 0.15 g/24 h [28]. “Clinical remission” referred to significant improvement in clinical symptoms and signs; quantitative reduction of urine protein≥50%; reduction of urine sediment erythrocyte count≥50%, or reduction of urine protein and urine occult blood≥“2+” [18, 28]. “Clinical cure rate” was defined as the number of clinical cures divided by the total number of participants. As well as definition of “partial remission rate” was the number of clinical remissions divided by the total number of participants. The sum of “clinical cure rate” and “partial remission rate” were “total effective rate”.

#### Exclusion criteria

(1) Participants included in RCTs had serious complications or other serious diseases (autoimmune disease, connective tissue disease, hemopathy, tumor, liver disease, heart failure, myocardial infarction, infectious disease, organ transplants and so on). (2) Participants included in RCTs received hemodialysis or peritoneal dialysis treatment. (3) Any Chinese herbal medicine preparations (oral and topical) other than HQH in the study, including single herbs, compound herbal medicines, herbal extracts, raw materials, Chinese patent herbal medicines and herbal formulas prescribed by practitioners. (4) Unable to acquire full text of the publications.

### Search strategy

We searched the following eight Chinese and English databases from their inception to December 2020. Four Chinese databases included China National Knowledge Infrastructure (CNKI), Wan Fang, Chinese Science and Technology Journal Database (VIP), and SinoMed Database. Four English databases included PubMed, EMBASE, the Cochrane Library, and Web of Science. Two trial registers including Clinical Trials. gov and the World Health Organization International Clinical Trials Registry Platform were also searched. Additionally, related reviews, conference papers, references lists and gray literatures also were searched manually to minimize the missed detection rate. No language or publication type was imposed. Taking “PubMed” as an example, the retrieval strategy was as follows: #1: ‘Purpura, Schoenlein-Henoch’[Mesh], #2: ‘Nephritis’[Mesh], #3: #1 AND #2, #4: ‘Henoeh-Sehonlein purpura nephritis’, #5: ‘purpura nephritis’, #6: ‘HSPN’,#7: ‘Immunoglobulin A vasculitis with nephritis’, #8: ‘IgAVN’, #9: #3 OR #4 OR #5 OR #6 OR #7 OR #8, #10: ‘child’[Mesh], #11: ‘children’, #12: #10 OR #11,#13: #9 AND #12, #14: ‘Huaiqihuang’, #15: ‘HQH’, #16: #14 OR #15, #17: #13 AND #16.

### Studies selection and data extraction

The titles and the abstracts were screened first, then the full-text versions were checked according to inclusion and exclusion criteria. Two authors examined the full text to identify the eligible trials independently. We made a data extraction sheet in advance. Two evaluators extracted data independently. Disagreements of studies selection and data extraction were resolved by the corresponding author JP Liu. Information was extracted from each included trial on: (1) basic characteristics of the literature, including the first author, publication year, sample size of each group, intervention (including types of intervention; names, doses, duration and frequency of HQH). (2) methodological characteristics of the trials. (3) types of primary and secondary outcome measure, length of follow up and adverse events reported.

### Quality assessment

#### Risk of bias in included RCTs

Two authors assessed and validated the quality of included trials independently according to Cochrane Handbook for Systematic Reviews of Interventions [29]. Seven items including random sequence generation, allocation concealment, blinding of participants and personnel, blinding of outcome assessment, incomplete outcome data, selective reporting and other bias (pharmaceutical funding and comparability of baseline data of subjects between groups), were used to be judged as “unclear risk”, “low risk”, or “high risk”. Any disagreements were resolved by discussion with the corresponding author JP Liu.

#### Certainty of evidence for main findings

Two reviewers summarized the certainty of evidence for main findings using the “Grading of Recommendations Assessment, Development, and Evaluation (GRADE) approach”, independently. The certainty of evidence for the main findings was graded as GRADE working Group grades of evidence. “High certainty”: very confident that the true effect size was close to the effect estimate. “Moderate certainty”: have a moderate degree of confidence in the estimated value of effect; the true value may be close to the estimated, but there was still the possibility that the two were significantly different. “Low certainty”: limited confidence in the estimated value of the effect; the true value may be very different from the estimated. “Very low certainty”: there was little confidence in the effect estimate, and the true effect was likely to be very different from the estimated [30].

### Statistical analysis

RevMan 5.3 software was used for data analysis. For continuous data, we used mean difference (MD) with 95% confidence intervals (CI) or standardized mean difference (SMD) with 95% CI (When the measurement unit of outcome was different, in order to eliminate the influence of the dimension, SMD can be selected). And for dichotomous data, we used risk ratio (RR) with 95% CI. We performed meta-analyses for trials if the study design, interventions, control and outcome measures were similar. Other data not suitable for pooling analysis were synthesized qualitatively.

I-square (I^2^) was used to test the statistical heterogeneity as recommended by the Cochrane Handbook for Systematic Reviews of Interventions [31]. A rough interpretation of I^2^ statistic value in the context of meta-analyses of randomized trials was as follows: 0 to 40%: might not be important; 30 to 60%: may represent moderate heterogeneity; 50 to 90%: may represent substantial heterogeneity; 75 to 100%: considerable heterogeneity. To reduce clinical heterogeneity, we conducted subgroups analyses of different interventions. We used random effects model for data pooling with moderate statistical heterogeneity (if I^2^>30%), otherwise a fixed effect model was applied if the trials were basically homogeneous (if I^2^ ≤ 30%). In case of the data were available, we did sensitivity analysis to explore the robustness of results. And we did funnel plots to test for the possibility of publication bias if there were enough studies (generally, more than 10 trials).

## Results

### Search results

**Fig. 1:**
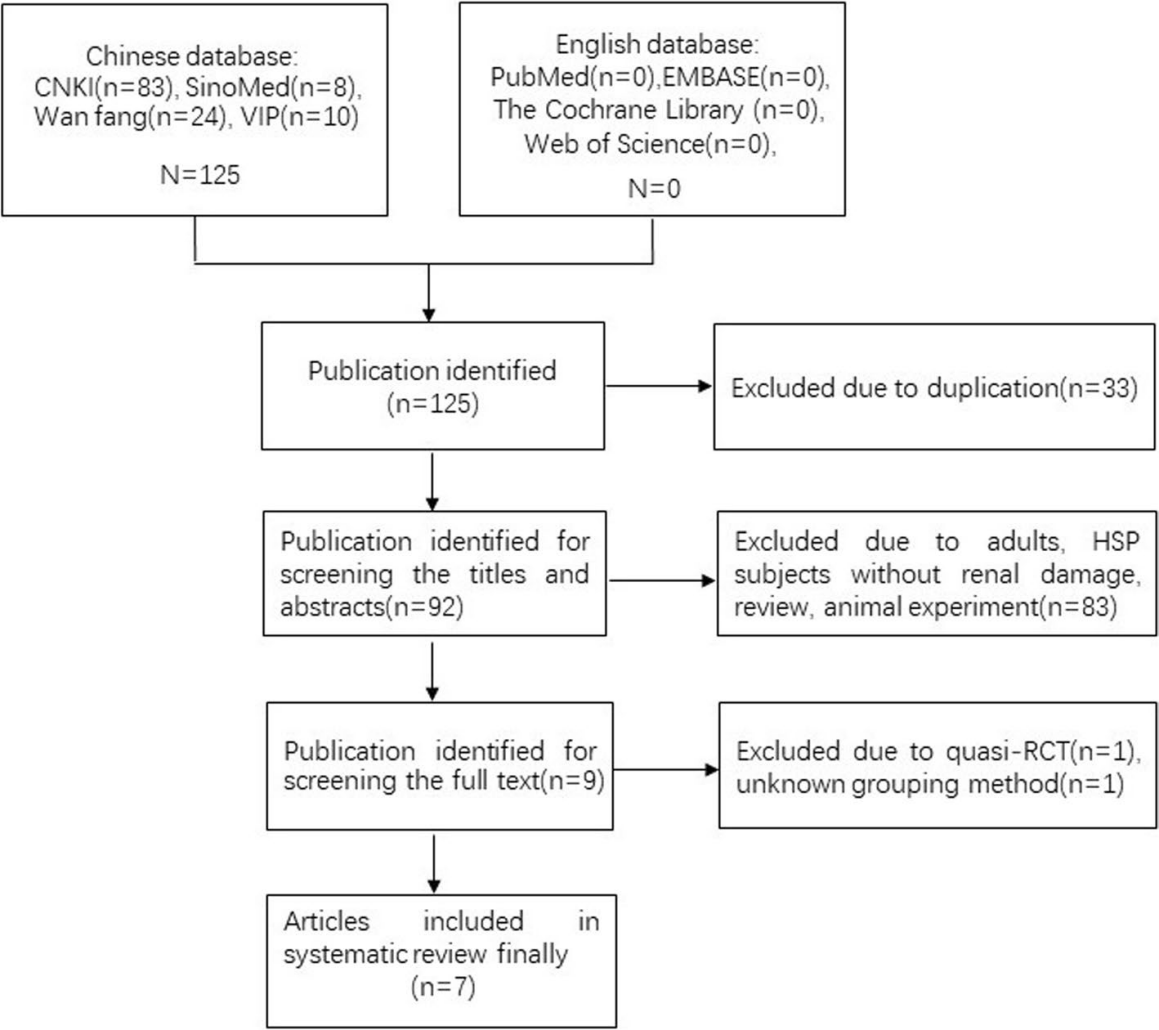
Flow diagram of study search and selection

One hundred and twenty-five relevant citations were retrieved from the searches. After scanning the full texts, two papers were excluded. In Wang N’s article [32], the ‘randomly allocate’ was grouped according to the order of visits. Single numbers were the HQH group, and double numbers were conventional treatment group. And in study of Ma XL [33], the grouping method was unknown. Finally, seven papers were eligible for this review. All of them were published in Chinese. Details of search and selection process are illustrated in Fig. 1.

### Characteristic of included trials

A total of seven RCTs involving 393 participants were included [13–19]. There was no incidence of end-stage renal disease, hemodialysis, peritoneal dialysis or mortality reported among all the trails. Among them, Zhou DJ’s citation was a stratified randomized controlled trial [19]. According to the random number table, 20 children with HSPN who were pathologically diagnosed as grade II were randomly divided into “basic treatment (BT)” group and “BT plus HQH” group, with 10 cases in each group. Also according to the random number table, 10 children with grade IIIa pathological examination were randomly divided into “GC” group and “GC plus HQH” treatment group, with 5 cases in each group. We used “Zhou DJ a” and “Zhou DJ b” to represent respectively. The main characteristics of included trials were demonstrated in Table 2.

**Table 2:** Characteristics of included trials on Huaiqihuang for Henoch-Schönlein purpura nephritis

Study ID	Sample size (E/C)	Age(years) (E/C)	Sex (Male) (E/C)	Clinical and pathological characteristics of samples	Intervention (on basis of BT)		Dosage and frequency of HQH	Duration of treatment	Follow-up	Outcomes
					E	C				
Duan CR 2017 [13]	36/36	6.34 ± 2.55/6.38 ± 2.54	23/22	Isolated hematuria/proteinuria; hematuria with proteinuria.	GC + HQH	GC	10 g/time, Bid.	4 weeks	12 weeks	①②⑩
Han DX 2014 [14]	38/35	Total 7.3 ± 2.4	Total 42	NR	GC + IS+HQH	GC + IS	≤3 years old, 5 g/time, Bid; >3 years old, 10 g/time, Bid.	8 weeks	NR	①②③④⑩
Lu W 2015 [15]	20/20	8.71 ± 5.02/8.12 ± 4.35	12/9	NR	GC + HQH	GC	≤5 years old, 5 g/time, Bid; >5 years old, 10 g/time, Bid.	1 month	NR	①②⑦⑧⑩
Peng YH 2014 [16]	28/26	7.15 ± 4.79/7.10 ± 5.12	16/15	Isolated hematuria/proteinuria; hematuria with proteinuria.	GC + HQH	GC	≤3 years old, 5 g/time, Bid; >3 years old, 10 g/time, Bid..	3 months	NR	①②⑧
Shi Z 2019 [17]	15/15	8.89 ± 1.67/8.45 ± 1.74	9/8	NR	GC + IS+HQH	GC + IS	10 g/time, Bid.	3 months	NR	①②③④⑤⑦⑩
Yuan TT 2020 [18]	47/47	8.6 ± 2.5/ 8.5 ± 2.6	31/33	Isolated hematuria/proteinuria; hematuria with proteinuria; III-IV pathological grades.	GC + IS+HQH	GC + IS	2–3 years old, 5 g/time, Bid; > 3 years old, 10 g/time, Bid.	3 months	NR	①②③④⑤⑧
Zhou DJ a 2013 [19]	10/10	Childen under 14 years	NR	II pathological grade.	HQH	BT alone	10 g/time, Bid.	2 months	NR	③④⑥⑨
Zhou DJ b 2013 [19]	5/5	Childen under 14 years	NR	IIIa pathological grade.	GC + HQH	GC	10 g/time, Bid.	2 months	NR	③④⑥⑨

*Abbreviations*: *HQH* Huaiqihuang, *E* Experimental group, *C* Control group, *NR* Not reported, *BT* Basic treatment, *GC* Glucocorticoid, *IS* Immunosuppressant, *+* Plus, *Bid* Two times a day, *Treg* Regulatory T cell, *Th17* T helper cell 17

①clinical cure rate; ②total effective rate; ③urinary protein; ④urine sediment erythrocyte count; ⑤urine β2 micro-globulin; ⑥levels of Treg and Th17; ⑦Concentration of IL-6; ⑧Concentration of IL-4; ⑨Concentration of IL-17; ⑩adverse events

### Risk of bias of included trials

**Fig. 2:**
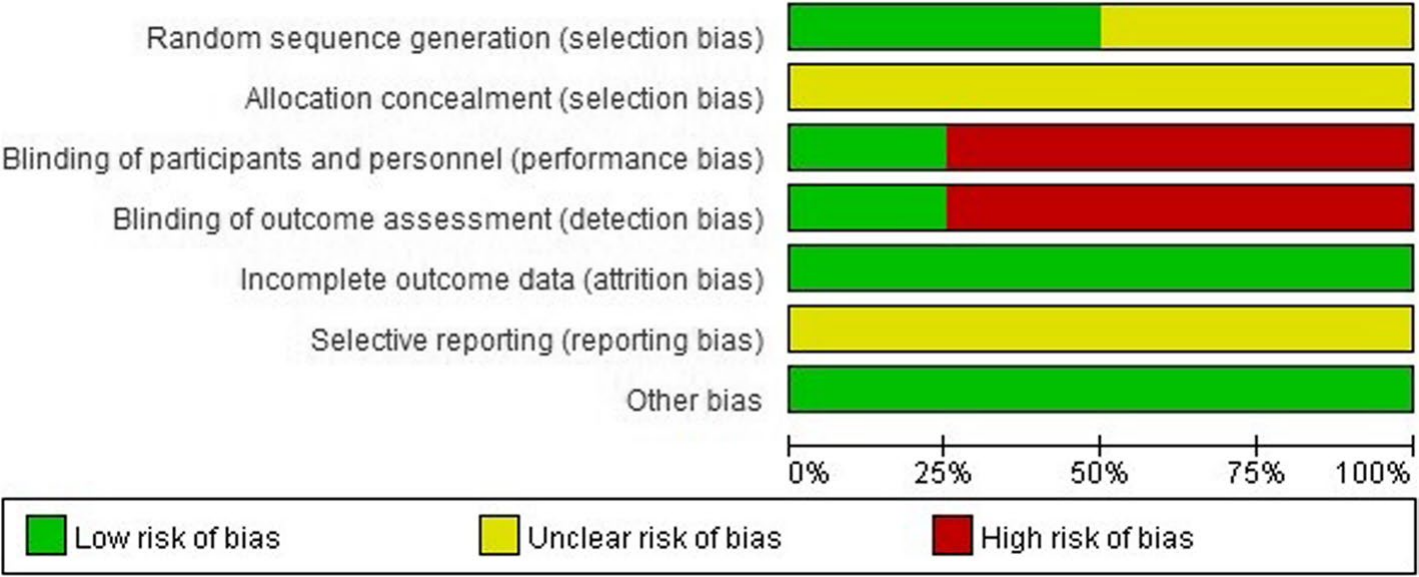
Risk of bias graph: review authors’ judgements about each risk of bias item presented as percentages across all included trials

All trials indicated “random allocation”. Three trials used the random number table [17–19], the remaining RCTs did not explain specific random methods [13–16]. The trials only describing “randomized allocation” were defined as “unclear risk” of bias, while the trials reporting a specific method of randomized sequences generation as “low risk”. No study provided sufficient information about allocation concealment. And “allocation concealment” of all trials were defined as “unclear risk” of bias. In addition, regarding blinding methods, no study used placebo granules. When the outcome measures of the study were only objective laboratory indicators, we defined performance bias and detection bias as “low risk”, as well as when the outcomes of the study included subjective indicators, we defined as “high risk”. No trial reported the number of participants lost to follow-up, so attrition bias was considered to be “low risk”. As the research protocol of each study was not obtained, we regarded reporting bias as “unclear risk” at last. All included studies did not receive support from pharmaceutical funding and no statistical difference was found in baseline characteristics between groups, so we considered other biases to be “low risk”. Despite we tried our best to contact authors by email or phone, no more information was obtained. In short, the included trials were evaluated for low methodological quality using a risk of bias assessment tool (Fig. 2).

### Outcome data and effects of interventions

#### Primary outcome measures

##### Clinical cure rate

**Fig. 3:**
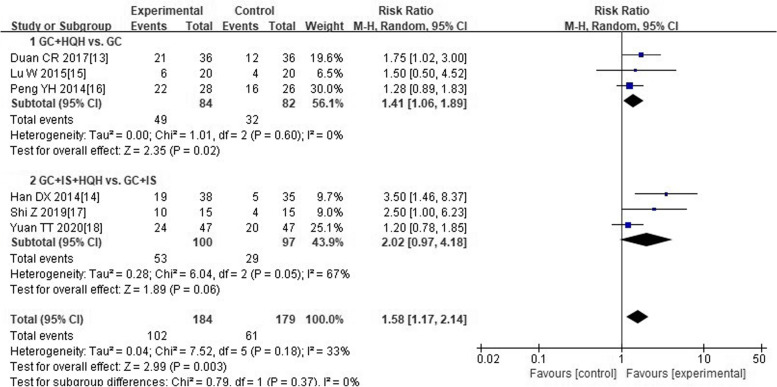
Forest plot of comparison on clinical cure rate between experimental group and control group. Abbreviations: GC: glucocorticoid; HQH: huaiqihuang; IS: immunosuppressant; +: plus

Six trials [13–18] reported clinical cure rate. Among them, 184 cases in experimental group, and 179 cases in control group. As presented in Fig. 3, the forest plot showed that compared with children treated with “conventional medicine” alone, children treated with “HQH combined with conventional medicine” had a more significant increase in clinical cure rates (RR 1.58; 95%CI 1.17 to 2.14). The statistical heterogeneity of each study was moderate (I^2^ = 33%). And taking into account the clinical heterogeneity of different interventions, we used random effect model. Additionally, we divided into two subgroups according to different conventional medicine interventions. Meta-analysis results of subgroups demonstrated that clinical cure rate of “GC plus HQH” group was better than “GC” group (RR 1.41; 1.06 to 1.89). It was not found that clinical cure rate of “GC plus IS plus HQH” group was higher than that of “GC plus IS” group (RR 2.02; 0.97 to 4.18).

##### Total effective rate

**Fig. 4:**
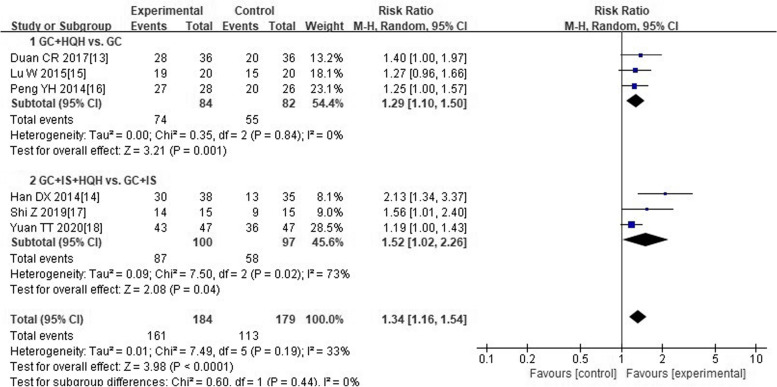
Forest plot of comparison on total effective rate between experimental group and control group. Abbreviations: GC: glucocorticoid; HQH: huaiqihuang; IS: immunosuppressant; +: plus

Six trials [13–18] reported total effective rate. Among them, 184 cases in experimental group, and 179 cases in control group. As shown in Fig. 4, the forest plot pointed that compared with children treated with “conventional medicine” alone, children treated with “HQH combined with conventional medicine” had a more obvious improvement in total effective rates (RR 1.34; 1.16 to 1.54). The statistical heterogeneity of each study was moderate (I^2^ = 33%). And taking into account clinical heterogeneity of different interventions, we applied random effect model. In addition, we divided into two subgroups according to different conventional medicine interventions. Meta-analysis results of subgroups indicated that total effective rate of “GC plus HQH” group was better than “GC” group (RR 1.29; 1.10 to 1.50). And children treated with “GC plus IS plus HQH” also suggested a more obvious elevation in total effective rates when compared with “GC plus IS” (RR 1.52; 1.02 to 2.26).

#### Secondary outcome measures

##### Urinary protein level

Four trials [14, 17–19] compared the effectiveness on reducing urinary protein between experimental and control group. Among them, studies of Shi Z [17] and Zhou DJ [19] reported the urinary albumin excretion (mg/L and mg/kg), all the others [14, 18] reported 24 h urinary protein (g/24 h). Due to different measurement units and considerable heterogeneity after pooling all results (I^2^ = 90%), we abandoned the overall pooling. We reported the result of each trial separately. In the trials of Han DX [14], Shi Z [17] and Yuan TT [18], additional administration of HQH had a superior effectiveness compared to “GC plus IS” on reducing proteinuria, with a MD (and 95%CI) of − 3.52 (− 4.26 to − 2.77); − 1.70 (− 2.55 to − 0.85) and − 0.92(− 1.35 to − 0.49), respectively.

In Zhou DJ’s trial [19], there was no significant statistical difference between “HQH” group and “BT” group in lowering proteinuria, with a MD of − 1.28, and 95%CI (− 2.83 to 0.27). While, “HQH plus GC” had a better effectiveness compared to “GC” group, with a MD of − 6.05, and 95%CI (− 9.86 to − 2.24).

##### Urine sediment erythrocyte count

**Fig. 5:**
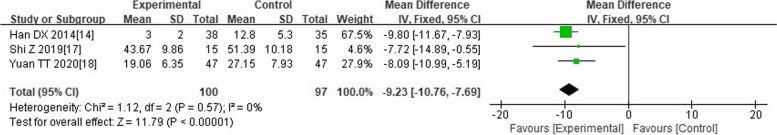
Forest plot of comparison on urine sediment erythrocyte count between experimental group (GC + IS+HQH) and control group (GC + IS). Abbreviations: GC: glucocorticoid; HQH: huaiqihuang; IS: immunosuppressant; +: plus

Four trials [14, 17–19] compared the effectiveness on reducing urine sediment erythrocyte count between the experimental and control group. Among them, trial of Zhou DJ [19] reported the urine sediment erythrocyte by unit of “× 10^7^ Pcs/L”, all the others [14, 17, 18] described by “Pcs/HP”. As a result of considerable heterogeneity after pooling all results (I^2^ = 95%), so we quitted the total pooling. We divided the different subgroups in accordance with different conventional medicine interventions. As shown in Fig. 5, the pooled result of 3 RCTs [14, 17, 18] manifested that additional administration of HQH had a superior effectiveness compared to “GC plus IS” on reducing urine sediment erythrocyte (MD -9.23; − 10.76 to − 7.69).

In Zhou DJ’s trial [19], there was no obvious statistical difference between “HQH” group and “BT” group in lowering urine sediment erythrocyte, with a MD of 0.31, and 95%CI (− 1.24 to 1.86). In addition, the results suggested “GC plus HQH” group had a superior effectiveness compared to “GC” group, with a MD of − 6.00, and 95%CI (− 9.22 to − 2.78).

##### Urineβ2 micro-globulin(β2-MG)

Two trials [17, 18] published urine β2-MG. There were 62 subjects in experimental group and 62 cases in control group. The unit of measurement for urine β2-MG in both two trials was “mg/L”. The pooled analysis indicated that “GC plus IS plus HQH” group had a better effect compared to “GC plus IS” group in decreasing urine β2-MG level (MD -0.09, − 0.12 to − 0.06). Heterogeneity of studies might be not important (I^2^ = 0%), and fixed effect model was selected.

##### Immune cells and inflammatory factors

**Levels of regulatory T cell (Treg) and T helper cell 17(Th17)** Zhou DJ’s trial [19] published outcomes of immune cells. The levels of Treg and Th17 were reported. The pooled result showed that compared with conventional medicine alone, co-intervention of HQH and conventional medicine had larger effect on elevating Treg (%) level (MD 5.02; 4.15 to 5.90). The statistical heterogeneity of each study might not be important (I^2^ = 0%). Considering the clinical heterogeneity, results of subgroups analyses were shown in Table 3.

**Table 3:** Subgroups analyses of different interventions on “immune cells and inflammatory factors” outcomes

Outcomes	Intervention (on basis of BT)		Sample size (E/C)	No. of studies	I^2^	Effect model	Effect estimation MD; (95% CI)	*P* value
	E	C						
Treg (%) [19]	HQH	BT alone	10/10	1 [19]	–	–	5.04; (4.15, 5.93)	*P*<0.00001
	GC + HQH	GC	5/5	1 [19]	–	–	4.42; (−1.20, 10.04)	*P* = 0.12
Th17 (%) [19]	HQH	BT alone	10/10	1 [19]	–	–	−0.89; (− 1.25, −0.53)	*P*<0.00001
	GC + HQH	GC	5/5	1 [19]	–	–	−0.32; (− 0.96, 0.32)	*P* = 0.33
IL-6 [15, 17]	GC + HQH	GC	20/20	1 [15]	–	–	−3.85; (−6.67, − 1.03)	*P* = 0.007
	GC + IS+HQH	GC + IS	15/15	1 [17]	–	–	−5.49; (−10.04, −0.94)	*P* = 0.02
IL-4 [15, 16, 18]	GC + HQH	GC	48/46	2 [15, 16]	61%	Random	−4.91; (−8.80, − 1.01)	*P* = 0.01
	GC + IS+HQH	GC + IS	47/47	1 [18]	–	–	−0.08; (− 0.11, − 0.05)	*P*<0.00001
IL-17 [19]	HQH	BT alone	10/10	1 [19]	–	–	− 14.06; (−21.74, − 6.38)	*P* = 0.0003
	GC + HQH	GC	5/5	1 [19]	–	–	−5.08; (−16.24, 6.08)	*P* = 0.37

*Abbreviations*: *Treg* Regulatory T cell, *Th17* T helper cell 17, *IL* Interleukin, *E* Experimental group, *C* Control group, *BT* Basic treatment, *GC* Glucocorticoid, *HQH* Huaiqihuang, *IS* Immunosuppressant, *+* Plus, *I*^*2*^ I-square, *MD* Mean difference, *CI* Confidence interval

Besides, the pooled result displayed that the function of lowering Th17(%) levels were more apparent after additional administration of HQH than conventional medicine alone (MD -0.75; − 1.07 to − 0.43). The heterogeneity of each study represented substantial heterogeneity (I^2^ = 56%). Thus, we performed subgroups analysis, and results were presented in Table 3.

**Concentration of Interleukin-6/Interleukin-4/Interleukin-17** Two trials [15, 17] reported concentration of interleukin 6 (IL-6), both of which were measured in “pg/mL”. The pooled result suggested that additional administration of HQH had a better effectiveness on decreasing IL-6 levels compared with “conventional medicine” alone (MD -4.30; − 6.70 to − 1.91). The heterogeneity of each study might not be important(I^2^ = 0%), and fixed effect model was adopted. A total of three trials [15, 16, 18] published concentration of interleukin 4 (IL-4). Among them, researches of Lu W [15] and Peng YH [16] both used “pg/mL” as the unit of measurement, while Yuan TT’s trial [18] adopted “μg/L” to measure IL-4. The pooled result of three trials showed that the effect of reducing IL-4 was more significant after adding HQH than conventional medicine alone (SMD -0.85; − 1.15 to − 0.54). The heterogeneity of each study might not be important (I^2^ = 4%), and fixed effect model was used. The concentration of IL-17 was described in citation of Zhou DJ [19], which was measured in “ng/L”. The pooled result of Zhou DJa and Zhou DJb indicated that the effect of reducing IL-17 was more significant after adding HQH than conventional medicine alone (MD -10.52; − 19.12 to − 1.92), with a moderate heterogeneity (I^2^ = 41%). Taking clinical heterogeneity into account, results of subgroups analyses were illustrated in Table 3.

##### Adverse events

**Fig. 6:**
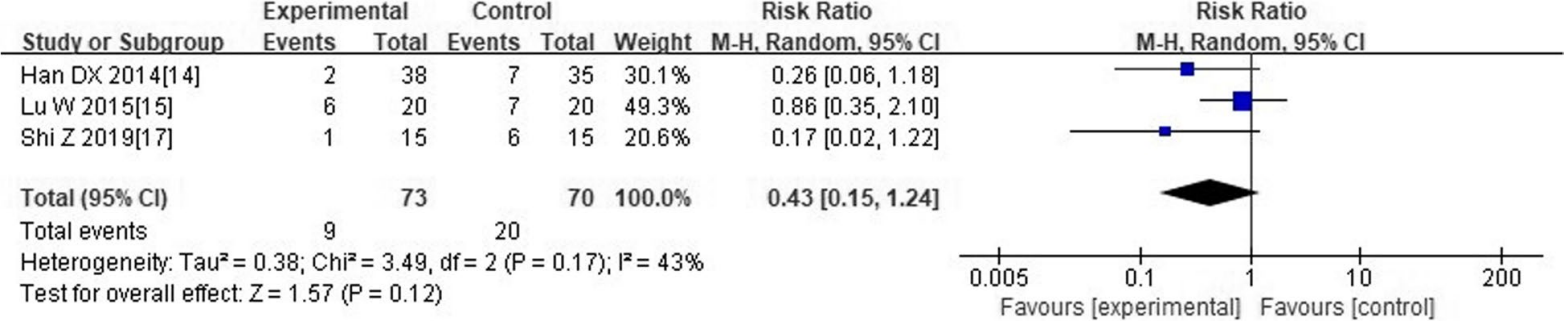
Forest plot of comparison on adverse events incidence between experimental group and control group

Only four trials [13–15, 17] reported the outcome of adverse events. Among them, one trial [13] described that there was no adverse event both in experimental group and control group throughout the whole study. Adverse events reported in the remaining three studies [14, 15, 17] were shown in Table 4. We did not acquire haematological biochemical record (blood routine, liver function) and any details about the treatment for adverse events from all included studies. None of the trials reported occurrence of serious adverse events such as permanent disability or death. As revealed in Fig. 6, the pooled analyses indicated that there was no significant statistical difference between “experimental group (co-intervention of HQH and conventional medicine)” and “control group (conventional medicine alone)” in incidence of adverse events, with an RR of 0.43 and a 95%CI (0.15 to 1.24).

**Table 4:** Adverse events of included trials on Huaiqihuang for Henoch-Schönlein purpura nephritis

Study ID	Sample size (E/C)	Adverse events cases	Experimental group (n)	Control group (n)
Duan CR 2017 [13]	36/36	E:0/36; C:0/36	Reported no occurrence	Reported no occurrence
Han DX 2014 [14]	38/35	E:2/38; C:7/35	Hypertension; secondary infection (Total 2)	Hypertension; secondary infection; mental symptoms (Total 7)
Lu W 2015 [15]	20/20	E:6/20; C:7/20	Adverse reaction of glucocorticoid (2) gastrointestinal discomfort (3) respiratory tract infection (1)	Adverse reaction of glucocorticoid (3) gastrointestinal discomfort (3) respiratory tract infection (1)
Peng YH 2014 [16]	28/26	E:NR; C:NR	NR	NR
Shi Z 2019 [17]	15/15	E:1/15; C:6/15	Diarrhea (1)	Itching(1), diarrhea(2), rash(1), nausea(1), dizziness (1)
Yuan TT 2020 [18]	47/47	E:NR; C:NR	NR	NR
Zhou DJ a 2013 [19]	10/10	E:NR; C:NR	NR	NR
Zhou DJ b 2013 [19]	5/5	E:NR; C:NR	NR	NR

*Abbreviations***:** *RCTs* Randomized controlled trials, *HQH* Huaiqihuang, *E* Experimental group, *C* Control group, *NR* Not reported

### Certainty of evidence for main findings

Combined with GRADE assessment results, the overall certainty of evidence for main findings were not good. Ranged from “very low” to “moderate”. In most cases, certainty of evidence was downgraded owing to serious limitations of primary studies on design and execution. Furthermore, as a result of fewer than 10 articles were included, no publication bias test was performed, we downgraded it by one level finally (Table 5).

**Table 5:** Summary of findings for the main comparisons in randomized controlled trials on Huaiqihuang for Henoch-Schönlein purpura nephritis

Certainty assessment							No. of patients		Effect		Certainty
No. of studies	Study design	Risk of bias	Inconsistency	Indirectness	Imprecision	Other considerations	E	C	Relative [95% CI]	Absolute [95% CI]	
Clinical cure rate 3 (GC + HQH versus GC)	RCT	Serious ^a^	Not serious	Not serious	Not serious	Publication bias unclear ^c^	49/84	32/82	RR 1.41 [1.06,1.89]	160 more per 1000 [from 23more to 347more]	⊕⊕⊖⊖ Low
Clinical cure rate 3 (GC + IS HQH versus GC + IS)	RCT	Serious ^a^	Serious ^b^	Not serious	Not serious	Publication bias unclear ^c^	53/100	29/97	RR 2.02 [0.97,4.18]	–	⊕⊖⊖⊖ Very Low
Total effective rate 3 (GC + HQH versus GC)	RCT	Serious ^a^	Not serious	Not serious	Not serious	Publication bias unclear ^c^	74/84	55/82	RR 1.29 [1.10,1.50]	195 more per 1000 [from 67more to 335more]	⊕⊕⊖⊖ Low
Total effective rate 3 (GC + IS+HQH versus GC + IS)	RCT	Serious ^a^	Serious ^b^	Not serious	Not serious	Publication bias unclear ^c^	87/100	58/97	RR 1.52 [1.02,2.26]	311 more per 1000 [from 12more to 753more]	⊕⊖⊖⊖ Very Low
Urine sediment erythrocyte 3 (GC + IS+HQH versus GC + IS)	RCT	Not serious	Not serious	Not serious	Not serious	Publication bias unclear ^c^	100	97	–	MD −9.23 [−10.76, −7.69]	⊕⊕⊕⊖ Moderate
Adverse events 3	RCT	Serious ^a^	Not serious	Not serious	Not serious	Publication bias unclear ^c^	9/73	20/70	RR 0.43 [0.15,1.24]	–	⊕⊕⊖⊖ Low

*Abbreviations*: *GC* Glucocorticoid, *HQH* Huaiqihuang, *IS* Immunosuppressant, *+* Plus, *RCT* Randomized controlled trial, *No.* Number, *E* Experimental group, *C* Control group, *CI* Confidence interval, *RR* Risk ratio, *MD* Mean difference

^a^ Random sequence generation, allocation concealment or blinding of the participants and personnel were poorly reported in over 50% of included studies; so certainty of evidence downgraded by one level

^b^ Heterogeneity is ‘serious’ and unexplained, thus, certainty of evidence downgraded by one level

^c^ Due to fewer than 10 articles were included, no publication bias test was performed. And we downgraded it by one level

As shown in Table 5, only few studies with small sample size included in the key clinical questions. Considering that small sample size of the included studies tends to exaggerate the effectiveness, the evidence may be not sufficient for clinical decision. We need to ensure enough power for the sample size estimation to enhance the precision and robustness of the result in future. As new studies may continue to be published, we should continue to update the systematic review on this topic, focusing on the above key clinical issues.

### Additional analysis

We explored the source of heterogeneity for subgroup whose heterogeneity was significant. Forest plot of subgroup between “GC plus IS plus HQH” group and “GC plus IS” group on “clinical cure rate” manifested substantial heterogeneity of each trial (Fig. 3, I^2^ = 67%). After removing the research of Han DX [14], heterogeneity among the trials reduced (I^2^ changed from 67 to 51%). As well as another forest plot of subgroup between “GC plus IS plus HQH” group and “GC plus IS” group on “total effective rate” showed substantial heterogeneity of trials (Fig. 4, I^2^ = 73%). Heterogeneity significantly declined (I^2^ changed from 73 to 21%) after excluding the trial of Han DX [14]. Considering that method of random sequence generation was unclear in Han DX’s trial [14], and two immunosuppressive agents were used in this trial, the factors of significant heterogeneity may be related to the above situation.

Furthermore, we finished meaningful sensitivity analyses. After eliminating the research of Han DX [14], sensitivity analyses did not change the direction of results, including outcomes of “clinical cure rate” (RR 1.55; 1.17 to 2.05) and “total effective rate” (RR 1.27; 1.13 to 1.42). The above suggested that results of the research could be consistent and robust.

Besides, since there was not enough (fewer than 10 articles) included trials, no publication bias test was performed.

## Discussion

### Principal findings and comparison with prior reviews

Although the literature has been comprehensively searched, we still have not found a systematic review on HQH’s intervention in HSPN. Thus, we evaluated the effectiveness and safety of HQH for HSPN in children, so as to provide evidence for clinical use. A total of seven trials were included in this study. In addition to pooling the overall results, we also did subgroups analyses according to different conventional medicine interventions in order to minimize clinical heterogeneity. Whether it was experimental group or control group, basic treatments were necessary. The application of GC or IS was based on clinical manifestation and pathological classification of the children. IS mainly included cyclophosphamide and mycophenolate mofetil. The results of our study demonstrated that compared with conventional medicine alone, the combined treatment of HQH and conventional medicine presented a superior effect in children with HSPN, including improving the clinical cure rate and total effective rate; reducing urinary protein excretion, urine sediment erythrocyte count and urine β2-MG level. With regard to subgroup analysis of “clinical cure rate”, despite the difference in cure rate between the two groups (“GC plus IS plus HQH” versus “GC plus IS”) was not statistically significant, it was still necessary to be cautious when drawing conclusions. Perhaps because of small sample size, the test power was not enough to find the difference between the two groups. In general, additional administration of HQH appeared to strengthen the protection of glomerulus and tubules in children with HSPN.

Moreover, many researches have confirmed that immune abnormalities and inflammatory injury played a key role in pathogenesis of HSPN recently [34, 35]. CD4^+^ T cells are important cells involved in the immune response. Tregs and Th17 cells, which belong to CD4^+^ T cell subsets, maintain the body’s immune balance. Tregs are responsible for the maintenance of self-tolerance, thus inhibiting autoimmunity, whereas pro-inflammatory Th17-cells contribute to the induction and propagation of inflammation [36]. In children with HSPN, an imbalance of Th17/Treg cell axis has been observed, which is often manifested as a decrease in Treg level and an increase in Th17 [37]. IL-17 is an important cytokine of Th17, which exerts immuno-suppressive effects [38]. IL-6 is an essential cytokine for the differentiation process of Th17 cells [39], and IL-4 is also the main pro-inflammatory factor [18]. Relevant studies have shown that the above-mentioned inflammatory factors were significantly elevated in children with HSPN. The pooling results of our study indicated that compared with conventional medicine alone, additional administration of HQH had better effect on elevating Treg (%), lowering Th17 (%) as well as lowering concentration of IL-6, IL-4 and IL-17. However, subgroups analyses results showed that compared with the “GC” group, co-intervention of HQH and GC did not have an advantage in increasing Treg and decreasing Th17 and IL-17. Maybe GC had powerful function of inhibiting the inflammatory factor IL-17, while the effect of HQH was masked. But considering extremely sparse sample size, the results need to be further confirmed in the future. Generally speaking, HQH seemed to regulate immune function and reduce inflammation damage in children with HSPN.

In terms of safety, the adverse events reported might be related to the side effects of medications under investigation. Adverse events observed mainly included hyper-tension, secondary infection, mental symptoms, gastrointestinal discomfort and so forth. In most cases, the reported abnormal symptoms were mild. There were no reports of serious adverse events in all the included studies. Combined with pooled results of meta-analyses, additional administration of HQH did not seem to increase the incidence of adverse events.

### Limitations

We conducted a systematic searching and appraised the original studies critically. In spite of there were lots of trials on HQH treating HSP or HSPN, only seven trials met our inclusion criteria. Due to generally poor methodological quality of included trials and some outcome measures were mentioned in only one article, there was no richer evidence to support the results. In addition, there were certain differences in the types of immunosuppressants, the dosage and frequency of HQH administration, as well as the duration of treatment in included studies. Meanwhile, the literature generally did not report follow-up and relapse, and it was impossible to evaluate the long-term effectiveness, which may affect the reliability of the results. Apart from this, all the trials were carried out in China, so the limitation on the race of patients need to be considered. At last, there was not enough included trials, no publication bias test was performed. All the above limited the overall confidence of evidence, and our result may need to be explained with caution.

### Implications for further research

As for GRADE assessment results, the overall certainty of evidence for main findings ranged from “very low” to “moderate”. Consequently, highly quality RCTs with better design are necessary in the future to improve the level of evidence. Furthermore, considering the above limitations, it is also important to evaluate the long-term effects and safety of interventions in future studies. So adequate follow-up time is required to observe relapse and adverse events.

## Conclusions

Chinese patent herbal medicine HQH combined with conventional medicine presented the beneficial effect for children with HSPN. And additional administration of HQH did not seem to increase the incidence of adverse events. However, considering the limited included studies and poor methodological quality, we should be cautious when using evidence in clinical practice. In the future, long term follow-up, high quality and multicenter RCTs are required to make the results more convincing.

## Data Availability

All data in our current study came from publicly available databases, including eight Chinese and English databases. And details related to this article are available from the corresponding author upon reasonable request.

## Abbreviations

HSP: Henoch-Schönlein purpura
HSPN: Henoch-Schönlein purpura nephritis
ACEIs: Angiotensin-converting enzyme inhibitors
ARBs: Angiotensin receptor blockers
GC: Glucocorticoid
IS: Immunosuppressant
HQH: Huaiqihuang
RCTs: Randomized controlled trials
ISKDC: International Study of Kidney Disease in Children
BT: Basic treatment
β2-MG: β2 micro-globulin
CNKI: China National Knowledge Infrastructure
VIP: Chinese Science and Technology Journal Database
IgAVN: Immunoglobulin A vasculitis with nephritis
GRADE: Grading of Recommendations Assessment, Development, and Evaluation
MD: Mean difference
CI: Confidence interval
SMD: Standardized mean difference
RR: Risk ratio
I^2^: I-square
Pcs/HP: Pieces/High-power field
Treg: Regulatory T cell
Th17: T helper cell 17
IL: Interleukin
E: Experimental group
C: Control group
NR: Not reported
+: Plus
Bid: Two times a day
